# Cancer development and mortality differences in patients with glomerulonephritis after renal biopsy: a single center retrospective cohort study

**DOI:** 10.1186/s12882-020-01882-x

**Published:** 2020-06-10

**Authors:** Hyunjin Ryu, Kipyo Kim, Jiwon Ryu, Hyung-Eun Son, Ji-Young Ryu, Sejoong Kim, Ki Young Na, Dong Wan Chae, Ho Jun Chin, Korean GlomeruloNephritis Study Group

**Affiliations:** 1 Department of Internal Medicine, Seoul National University Hospital, Seoul, Republic of Korea; 2 Department of Internal Medicine, Inha University Hospital, Incheon, Republic of Korea; 3 Department of Internal medicine, Cheju Halla General Hospital Cheju, Republic of Korea; 4 Department of Internal Medicine, Seoul National University Bundang Hospital, Seongnam, Republic of Korea; 5 Department of Internal Medicine, Seoul National University College of Medicine, Seoul, Republic of Korea

**Keywords:** Glomerulonephritis, de novo cancer, Mortality, Outcome

## Abstract

**Background:**

The association between glomerulonephritis (GN) and cancer has been well known for decades. However, studies evaluating long-term de novo cancer development in patients with GN are limited. This study aimed to evaluate the incidence of cancer development among patients with renal biopsy-proven GN during post-biopsy follow-up and the differences in outcomes according to cancer occurrence.

**Methods:**

We conducted a retrospective cohort study of adult patients who underwent renal biopsy at Seoul National Bundang Hospital between 2003 and 2017. After excluding 778 patients with age < 18 years, cancer diagnosis before or within 6 months after renal biopsy, immunosuppressant therapy before renal biopsy, or pathologic diagnoses other than GN, 822 patients were included in the analysis. Data on baseline clinical characteristics, renal biopsy results, and types and doses of immunosuppressant agents were collected from electronic medical records. The incidence of cancer was censored on the date when the first cancer was diagnosed. We evaluated rates of mortality and end-stage renal disease (ESRD) development during follow-up.

**Results:**

During a mean follow-up period of 58.9 ± 44.5 months, 45 subjects (5.5%) developed de novo cancer. A comparison of clinical characteristics between subjects who did and did not develop cancer revealed that cancer patients were older and had higher comorbidities and immunosuppressant use. Overall, patients with GN had an elevated standardized incidence ratio (SIR) of 7.16 (95% confidence interval (CI): 5.22–9.61) relative to the age- and sex-matched general population. In particular, the SIR was significantly higher in GNs such as membranous nephropathy (MN), IgA nephropathy, lupus nephritis, and focal segmental glomerulosclerosis. Multivariable Cox proportional hazard model revealed that patients with MN had an increased risk of cancer development, with a hazard ratio of 2.30 [95% CI: 1.06–4.98]. Patients with MN who developed cancer had a significantly higher risk of mortality (hazard ratio: 6.59; 95% CI: 1.22–35.56, *P* = 0.03) than those without cancer, but there was a non-significant difference in ESRD development. **Conclusions:** Patients with GN without concurrent cancer, particularly those with MN, have significantly higher risks of cancer development and subsequent mortality and should remain aware of the potential development of malignancy during follow-up.

## Background

Glomerulonephritis (GN) is a heterogeneous set of diseases caused by immune or inflammatory pathogenesis in the glomerular lesion. Although the incidence of GN is decreasing worldwide, it remains the third leading cause of end-stage renal disease (ESRD) in most countries, including South Korea [1–3]. The incidence of GN exhibits two peaks for age at the age of 20–30 years and ≥ 60 years [4]. In older adults, membranous nephropathy (MN) is the major type of GN, which is associated with a 5-fold higher prevalence of malignancy relative to the reference population [5]. This finding highlights the need to screen for malignancies in patients with GN, particularly in older patients, at the time of diagnosis and during the follow-up period [4, 6, 7].

The association of GN and cancer has been well known for more than half a century [8]. Various types of cancers including lung, skin, lymphatic, and hematologic malignancies are known to be associated with GN and its pathophysiology [9, 10]. In particular, associations have been identified between MN and solid tumors [9], between minimal change diseases (MCD) and Hodgkin’s lymphoma, and between membranoproliferative glomerulonephropathy (MPGN) and chronic lymphocytic leukemia [11, 12]. These associations have been attributed to mechanisms such as the production of tumor antigens, secretion of circulating factors by T lymphocytes, or production of cryoglobulin or M-component by B lymphocytes [13–15]. Therefore, the Kidney Disease Improving Global Outcomes guidelines recommend screening for underlying malignancy in patients diagnosed with MN, focal segmental glomerulosclerosis (FSGS), and immunoglobulin A nephropathy (IgAN) [16]. Moreover, there have been a few reports that have described an association between GN and the long-term incidence of cancer [10, 17, 18].

Patients with GN are treated and monitored mainly by nephrologists. Consequently, nephrologists must be aware of the long-term cancer incidence and outcomes among patients with GN. Previous studies on this topic were conducted in Western populations and focused mainly on MN or nephrotic syndrome and have not elucidated the effects of de novo cancer development on the mortality and renal outcomes of patients with GN. Therefore, in this retrospective cohort study, we analyzed the de novo cancer incidence of patients with biopsy-proven GN in the Korean population and attempted to identify risk factors for de novo cancer. Furthermore, we explored the effects of cancer on mortality and ESRD development using the longitudinal data in our cohort.

## Methods

### Study population

**Fig. 1:**
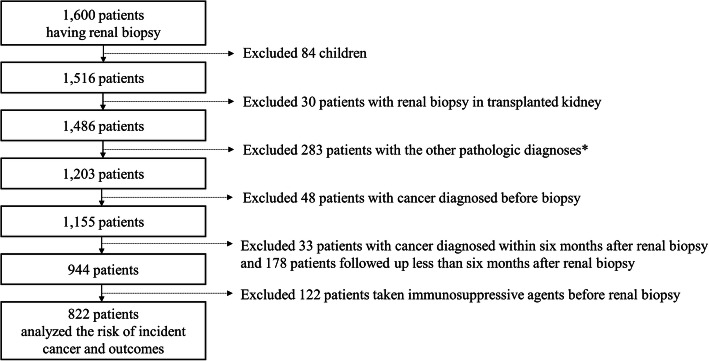
Flow chart of study subjects. * Pathologic diagnoses other than non-specific GN, amyloidosis, crescentic GN, diabetic nephropathy, focal segmental glomerulosclerosis, immunoglobulin A nephropathy, lupus nephritis, minimal change lesion, membranous nephropathy, membranoproliferative glomerulonephritis -immune complex type, tubulointerstitial nephropathy, or thrombotic microangiopathy

This single-center retrospective longitudinal cohort study was conducted at Seoul National University Bundang Hospital, a tertiary hospital facility. We initially identified 1600 patients who underwent renal biopsy between 2003 and 2017 at this hospital. Renal biopsies were conducted using the ultrasonography-guided percutaneous gun biopsy technique, and all specimens were initially evaluated by renal pathologists. The present study included patients with a pathological diagnosis of non-specific GN, amyloidosis, crescentic GN, diabetic nephropathy, FSGS, IgAN, lupus nephritis, MCD, MN, MPGN-immune complex type, tubulointerstitial injury (TIN), or thrombogenic microangiopathy (TMA). Patients who met any of the following criteria were excluded: any other renal pathologic diagnosis, age < 18 years, history of renal transplantation, medical history of cancer (diagnosed before renal biopsy) or concurrent cancer (diagnosed within 6 month of renal biopsy), loss to follow-up within 6 month of biopsy, or a history of immunosuppressive agent therapy before renal biopsy. After excluding 778 patients, 822 adult patients were included in the retrospective longitudinal analysis (Fig. 1).

### Data collection

The electronic medical records of the study population were reviewed retrospectively to obtain the following data for the longitudinal study. Baseline clinical information at the time of renal biopsy, including age, sex, prior medical history, reason for biopsy, and systolic and diastolic blood pressure, were collected. Laboratory results such as the serum protein, albumin, bilirubin, cholesterol, glucose, hemoglobin, creatinine, spot urine protein–creatinine ratio, and albumin by dipstick test (defined as ≥2 positive) were also collected. The pathologic results and diagnosis from the renal biopsy were evaluated. The estimated glomerular filtration rate (eGFR) was calculated using the Chronic Kidney Disease-Epidemiology Collaboration equation [19]. Additional data were obtained during the follow-up after biopsy, including the types and doses of immunosuppressant agents such as azathioprine, cyclophosphamide, mycophenolate, rituximab, prednisolone, tacrolimus, and cyclosporine. Data related to the primary study outcome of a cancer diagnosis and the secondary outcomes of mortality and renal replacement therapy during follow-up were also collected. The incidence of cancer was defined at the time of the initial cancer diagnosis and the first use of any International Statistical Classification of Diseases and Related Health Problems, 10th revision (ICD-10) code, ranging from C00 to D48, during follow-up. In South Korea, most of the medical expenditures incurred by a patient with a registered cancer diagnosis are covered by the government-issued universal health insurance system. Therefore, cancer diagnosis-related ICD-10 codes of patients who were diagnosed with cancer at other hospitals were also registered at our hospital to ensure that their care would be covered by the expanded insurance benefits. Information on mortality during follow-up was determined through both a review of the electronic medical records and a search of the National Health Insurance System and Korea Statistical Information Service databases for participants who were lost to follow-up. Cases requiring renal replacement therapy for a duration > 3 months during follow-up were detected using data from the ESRD registry-Korea Society of Nephrology [20]. The standardized incidence ratio (SIR) was calculated using the National Cancer Center (NCC) registry data to determine whether the incidence of de novo cancer was increased among the patients with GN relative to the general population. The NCC registry data provide cancer incidence data for the general Korean population collected during health screenings between 2003 and 2015, stratified by sex and 5-year age groups [21].

### Statistical analysis

All analyses were performed using IBM SPSS Statistics (Version 25.0, IBM, NY, USA). Categorical variables are expressed as numbers and percentages, and continuous variables are expressed as means ± standard deviations. The chi-square test and t-test were used to compare differences in baseline characteristics between subjects who did and did not develop cancer. Kaplan–Meier survival curves of cancer-free survival according to the pathologic diagnoses were compared using the log-rank test. Cox proportional hazard models were used to identify risk factors related to cancer development after renal biopsy. Model 1 was an unadjusted model for associations between pathologic diagnosis and cancer development. Model 2 was adjusted for age, sex, and clinical parameters such as a history of coronary heart disease, diabetes mellitus, chronic hepatitis B and C, liver cirrhosis, smoking history, hemoglobin, and serum creatinine levels at the time of renal biopsy. Model 3, the fully adjusted model, was additionally adjusted for the use of immunosuppressive agents such as azathioprine, cyclophosphamide, cyclosporine, mycophenolate, steroid, rituximab, and tacrolimus after renal biopsy but before cancer development. Subgroup analysis was conducted among MN patients using a Cox proportional hazard model adjusted for age, sex, serum sodium, hemoglobin concentrations, and pathologic findings such as global sclerosis and the presence of mesangial electron-dense deposits. The results of these analyses are presented as hazard ratios (HRs) with 95% confidence intervals (CIs). Two-sided *p-*values were reported with 0.05 taken as the level of statistical significance.

## Results

### Baseline characteristics of the study population

A total of 822 subjects were included in the final analysis. The mean age of included patients was 48.5 ± 15.9 years, 48.8% was male, and 7.8, 6.6, 16.0, and 61.8% had a history of cerebrovascular disease, coronary heart disease, diabetic mellitus, and hypertension, respectively. The mean eGFR was 79.6 ± 35.3 mL/min/1.73 m^2^. The final pathologic diagnoses of the subjects were nonspecific GN (*n* = 28, 3.4%), amyloidosis (*n* = 3, 0.4%), crescentic GN in 19 (2.3%), diabetic nephropathy in 51 (6.2%), FSGS in 82 (10.0%), IgAN in 366 (44.5%), lupus nephritis in 25 (3.0%), MCD in 69 (8.4%), MN in 111 (13.5%), MPGN in 32 (3.9%), TIN in 31 (3.8%) and TMA in 5 subjects (0.6%).

### Incidence of de novo cancers in the study population

Forty-five subjects (5.5%) were newly diagnosed with cancer during a mean follow-up duration of 58.9 ± 44.5 months. The most common malignancies were hepatocellular carcinoma (13.3%), colon cancer (11.1%), papillary thyroid carcinoma (8.9%), gastric cancer (6.7%), prostate cancer (6.7%), and lymphoma (6.7%). The numbers of patients with incident cancer and the types of cancer are presented according to the initial renal pathologic diagnosis in Table 1. The cancer incidence according to pathologic diagnosis and associated *p*-values are shown in Figure S1. The cancer incidence rates were significantly higher in MN (9.9%) and lower in IgAN (3.3%) compared to other pathologic diagnoses (*P* = 0.03 and *P* = 0.01, respectively). The age- and sex-standardized incidence ratio of de novo cancers in the cohort from 2003 to 2017 are presented in Table S1. The overall study cohort had an elevated SIR of 7.16 (95% CI: 5.22–9.61) relative to the age- and sex-matched general population. Additionally, when the SIR was calculated according to specific GN types, subjects with lupus nephritis (SIR: 39.46, 95% CI: 7.93–129.29), crescentic GN (SIR: 10.06, 95% CI: 1.13–43.68), MN (SIR: 8.92, 95% CI: 4.44–16.30), TIN (SIR: 8.82, 95% CI: 1.77–28.89), diabetic nephropathy (SIR: 8.31, 95% CI: 2.24–23.07), FSGS (SIR: 7.73, 95% CI: 2.49–19.17), and IgAN (SIR: 6.05, 95% CI: 3.12–10.77) had increased risks of cancer relative to the general population, although the lack of significant differences for other types of GN might have been due to the small numbers of analyzed subjects.

**Table 1:** Cancer incidence during the follow-up period after renal biopsy

	Nonspf	Amyl	CreGN	DMN	FSGS	IgAN	LN	MCD	MN	MPGN	TIN	TMA	Total
Total number of participants	28	3	19	51	82	366	25	69	111	32	31	5	822
Number of patients with incident cancer	1	1	2	4	5	12	3	1	11	2	3	0	45
Acute myelocytic leukemia	0	0	0	0	0	0	0	0	1	0	0	0	1
Acute myeloid leukemia	0	0	0	0	0	0	1	0	0	0	0	0	1
Bladder cancer	0	0	0	0	0	1	0	0	1	0	0	0	2
Breast cancer	0	0	0	0	0	1	0	0	0	0	0	0	1
Colon cancer	0	0	0	1	1	0	0	1	1	0	1	0	5
Hepatocellular carcinoma	0	0	0	3	0	1	0	0	1	1	0	0	6
Lung cancer	0	0	1	0	0	1	0	0	1	0	0	0	3
Lymphoma	0	0	0	0	0	0	1	0	1	0	1	0	3
Malignant brain tumor	0	0	0	0	1	0	0	0	0	0	0	0	1
Malignant neoplasm of retroperitoneum	0	0	0	0	0	0	0	0	0	0	1	0	1
Malignant neoplasm of soft tissue	0	0	0	0	1	0	0	0	0	0	0	0	1
Melanoma of rectum	0	0	0	0	0	1	0	0	0	0	0	0	1
Metastatic tumor to the spine	1	0	1	0	0	0	0	0	0	0	0	0	2
Multiple myeloma	0	1	0	0	0	0	0	0	1	0	0	0	2
Pancreatic cancer	0	0	0	0	0	0	0	0	0	0	0	0	0
Papillary thyroid carcinoma	0	0	0	0	0	4	0	0	0	0	0	0	4
Prostate cancer	0	0	0	0	1	1	0	0	1	0	0	0	3
Renal cell carcinoma	0	0	0	0	1	1	0	0	0	0	0	0	2
Squamous cell carcinoma of skin	0	0	0	0	0	0	0	0	2	0	0	0	2
Stomach cancer	0	0	0	0	0	1	0	0	1	1	0	0	3
Unspecified mass	0	0	0	0	0	0	1	0	0	0	0	0	1

*Nonspf* Nonspecific glomerulonephritis, *Amyl* Amyloidosis, *CreGN* Crescentic glomerulonephritis, *DMN* Diabetic nephropathy, *FSGS* Focal segmental glomerulonephritis, *IgAN* IgA nephropathy, *LN* Lupus nephritis, *MCD* Minimal change disease, *MN* Membranous nephropathy, *MPGN* Membranoproliferative glomerulonephritis, *TIN* Tubulointerstitial nephropathy, *TMA* Thrombotic microangiopathy

### Risk factors associated with Cancer occurrence

**Fig. 2:**
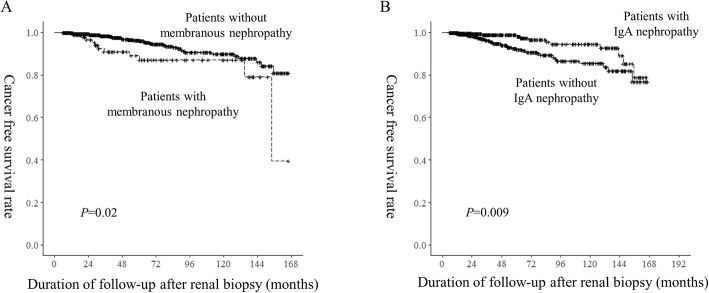
Kaplan-Meier survival curve for cancer-free survival in patients with or without membranous nephropathy (**a**) or IgA nephropathy (**b**). *P*-values were calculated by the log-rank test

**Fig. 3:**
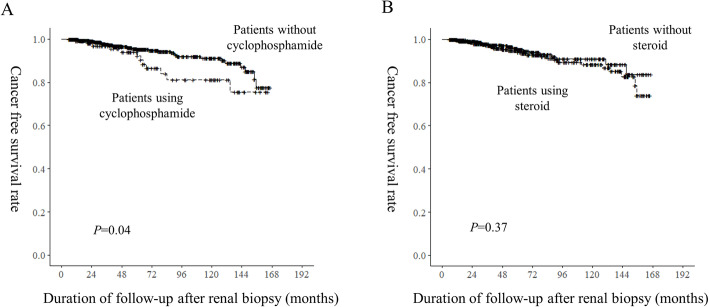
Kaplan-Meier survival curve for cancer-free survival according to the usage of cyclophosphamide (**a**) or steroid (**b**). *P*-values were calculated by the log-rank test

A comparison of the clinical characteristics between subjects who did and did not develop cancer during follow-up revealed that subjects with cancer were significantly older (57.1 ± 13.8 vs. 48.0 ± 15.9 years, *P* < 0.001) and had lower hemoglobin (11.8 ± 2.3 g/dL vs. 12.7 ± 2.2 g/dL, *P* < 0.001) and glucose levels (106.5 ± 30.5 vs. 118.6 ± 46.7, *P* = 0.02), and higher immunosuppressant use (62.2% vs. 43.0%, *P* = 0.02; Table 2). Moreover, when compared to subjects without cancer, those with cancer had more frequently used cyclophosphamide (26.7% vs. 13.6%, *P* = 0.03) and had received marginally higher cumulative doses of cyclophosphamide (5.35 ± 14.83 g vs 1.49 ± 5.56 g, *P* = 0.09). Kapan–Meier survival curves according to the pathologic diagnoses showed that patients with MN had a lower cancer-free survival rate than those without MN (*P* = 0.02; Fig. 2a), but those with IgAN had a higher cancer-free survival rate than those without IgAN (*P* = 0.009; Fig. 2b). Patients treated with cyclophosphamide showed a lower cancer-free survival rate than those not treated with cyclophosphamide (*P* = 0.04, Fig. 3a). We found no significant differences in the cancer-free survival rate with regard to steroid use (Fig. 3b) and other immunosuppressants (Fig S2). A multivariable Cox proportional hazard analysis was conducted to identify independent risk factors for cancer development (Table 3). The unadjusted model (Model 1) yielded an HR of 7.76 (95% CI: 1.06–57.05, *P* = 0.04), 2.27 (95% CI: 1.15–4.49, *P* = 0.02), and 0.43 (95% CI: 0.22–0.82, *P* = 0.01) for a pathologic diagnosis of amyloidosis, MN, and IgAN, respectively. However, after adjustment for relevant covariates, only MN showed significantly higher risks of cancer development among these diagnosis (Table S2). The fully adjusted model (Model 3) identified a pathologic diagnosis of MN (HR: 2.30, 95% CI: 1.06–4.98, *P* = 0.03), age (HR: 1.05, 95% CI: 1.02–1.07, *P* < 0.001), and hemoglobin (HR: 0.79, 95% CI: 0.67–0.94, *P* = 0.01) as significant risk factors for de novo cancer development.

**Table 2:** Clinical characteristics of patients according to the development of cancer after renal biopsy

	Cancer (−)	Cancer (+)	*P*-value
Number	777	45	
Age (year)	48.0 ± 15.9	57.1 ± 13.8	< 0.001
Gender (male, %)	376 (48.4)	25 (55.6)	0.44
Smoking history (n,%)	174 (24.4)	10 (23.3)	1.00
History of cerebrovascular disease (n, %)	60 (7.7)	4 (8.9)	1.00
History of coronary heart disease (n, %)	48 (6.2)	6 (13.3)	0.12
Diabetes (n, %)	118 (15.5)	11 (25.6)	0.12
Hypertension (n, %)	481 (61.9)	27 (60.0)	0.92
Chronic hepatitis B	29 (3.7)	3 (6.7)	0.55
Chronic hepatitis C	3 (0.4)	0 (0)	1.00
Liver cirrhosis (any cause)	16 (2.1)	1 (2.2)	1.00
Systolic blood pressure (mmHg)	128.3 ± 18.7	132.7 ± 23.9	0.24
Diastolic blood pressure (mmHg)	73.9 ± 11.9	73.1 ± 12.6	0.65
Protein (g/dl)	6.1 ± 1.1	6.2 ± 1.2	0.71
Albumin (g/dl)	3.4 ± 0.8	3.3 ± 0.7	0.25
Bilirubin (mg/dl)	0.5 ± 0.4	0.5 ± 0.2	0.93
Cholesterol (mg/dl)	217.1 ± 89.3	213.2 ± 74.2	0.79
Glucose (mg/dl)	118.6 ± 46.7	106.5 ± 30.5	0.02
Hemoglobin (g/dl)	12.7 ± 2.2	11.8 ± 2.3	< 0.001
Creatinine (mg/dl)	1.3 ± 1.3	2.0 ± 2.7	0.09
Glomerular filtration rate (ml/min/1.73 m^2^)	80.1 ± 35.2	70.3 ± 36.3	0.07
Urine protein to creatinine ratio (g/g cr)	2.9 ± 3.7	4.0 ± 4.4	0.07
Albumin by dipstick ≥2+ (n, %)	565 (76.0)	33 (75.0)	1.00
Pathologic diagnosis			0.05
Non-specific GN	27 (3.5)	1 (2.2)	
Amyloidosis	2 (0.3)	1 (2.2)	
Crescentic GN	17 (2.2)	2 (4.4)	
Diabetic nephropathy	47 (6.0)	4 (8.9)	
FSGS	77 (9.9)	5 (11.1)	
IgA nephropathy^a^	354 (45.6)	12 (26.7)	
Lupus nephritis	22 (2.8)	3 (6.7)	
MCD	68 (8.8)	1 (2.2)	
MN^a^	100 (12.9)	11 (24.4)	
Immune complex type of MPGN	30 (3.9)	2 (4.4)	
TIN	28 (3.6)	3 (6.7)	
TMA	5 (0.3)	0 (0.0)	
Follow up duration until the detection of cancer (months)	58.6 ± 44.6	63.4 ± 42.1	0.48
Usage of immunosuppressive medication until the detection of cancer			
Any kind of immunosuppressant (n, %)	334 (43.0)	28 (62.2)	0.02
Azathioprine (n, %)	35 (4.5)	4 (8.9)	0.33
Cyclophosphamide (n, %)	106 (13.6)	12 (26.7)	0.03
Mycophenolate (n, %)	49 (6.3)	4 (8.9)	0.71
Rituximab (n, %)	11 (1.4)	0 (0.0)	0.89
Prednisolone (n, %)	330 (42.5)	26 (57.8)	0.06
Tacrolimus (n, %)	79 (10.2)	3 (6.7)	0.61
Cyclosporine (n, %)	58 (7.5)	2 (4.4)	0.64
Total dose of immunosuppressive medication until detection of cancer			
Azathioprine (g)	1.71 ± 15.90	3.71 ± 16.09	0.41
Cyclophosphamide (g)	1.49 ± 5.56	5.35 ± 14.83	0.09
Mycophenolate (g)	63.65 ± 34.06	50.89 ± 27.94	0.81
Rituximab (g)	0.03 ± 0.30	0.0 ± 0.0	0.01
Prednisolone (g)	4.86 ± 10.78	6.95 ± 11.31	0.21
Tacrolimus (g)	0.20 ± 1.10	0.05 ± 0.27	0.01
Cyclosporine (g)	8.36 ± 50.04	1.11 ± 6.32	< 0.001

*GN* Glomerulonephritis; FSGS Focal segmental glomerulonephritis; *IgAN* IgA nephropathy; *MCD* Minimal change disease; *MN* Membranous nephropathy; *MPGN* Membranoproliferative glomerulonephritis; *TIN* Tubulointerstitial nephropathy; *TMA* Thrombotic microangiopathy

^a^ Statistically significant when the incidence of cancer was compared between patients with a certain pathologic finding and the others

**Table 3:** Risk factors related to cancer development after renal biopsy

	Variables	B	Wald	Hazard ratio	95% Confidence interval		*P*-value
Model 1	IgAN (presence)	− 0.86	6.43	0.43	0.22	0.82	0.01
	Amyloidosis (presence)	2.05	4.05	7.76	1.06	57.05	0.04
	MN (presence)	0.82	5.54	2.27	1.15	4.49	0.02
Model 2	MN (presence)	0.88	5.06	2.41	1.12	5.18	0.03
	Age (year)	0.05	14.06	1.05	1.02	1.07	< 0.001
	Hemoglobin (g/dl)	−0.24	7.71	0.79	0.66	0.93	0.01
Model 3	MN (presence)	0.83	4.42	2.30	1.06	4.98	0.03
	Age (year)	0.05	13.31	1.05	1.02	1.07	< 0.001
	Hemoglobin (g/dl)	−0.23	7.35	0.79	0.67	0.94	0.01

*IgAN* IgA nephropathy; *MN* Membranous nephropathy

Model 1: a univariate model for pathologic diagnosis by Cox’s hazard proportional model. Model 2: adjusted with age, gender, and clinical parameters related to the incidence of cancer, such as diabetes mellitus, coronary heart disease, smoking status, chronic hepatitis B and C, liver cirrhosis, and levels of hemoglobin and serum creatinine at renal biopsy. Model 3: adjusted with factors included in model 2 and usage of each immunosuppressive agent such as azathioprine, cyclophosphamide, cyclosporine, mycophenolate, steroids, rituximab, and tacrolimus after renal biopsy but before the development of cancer

### SIRs for specific Cancer types in patients with membranous nephropathy

Overall, MN patients showed a significantly higher age- and sex-standardized incidence ratio. Particularly, patients with squamous cell carcinoma of the skin, acute myelocytic leukemia, and multiple myeloma showed a significantly elevated SIR compared to the age- and sex-matched general population (Table S3). For other solid cancers, there were no significant differences in SIR.

### Risk factors for Cancer development in patients with membranous nephropathy

An additional multivariable Cox proportional hazard model was applied solely to subjects with MN to determine the independent risk factors for de novo cancer development. After adjusting for clinical parameters at the time of renal biopsy, such as age, sex, serum sodium, and hemoglobin levels as well as pathologic findings of global sclerosis and presence of mesangial electron dense deposits, only age was identified as a significant factor. Specifically, subjects with MN who were aged ≥65 years had significantly increased HRs of 7.61 (95% CI: 1.56–37.16, *P* = 0.01), when compared to patients aged < 65 years (Table S4).

### Effect of Cancer development on patients and Renal survival

**Fig. 4:**
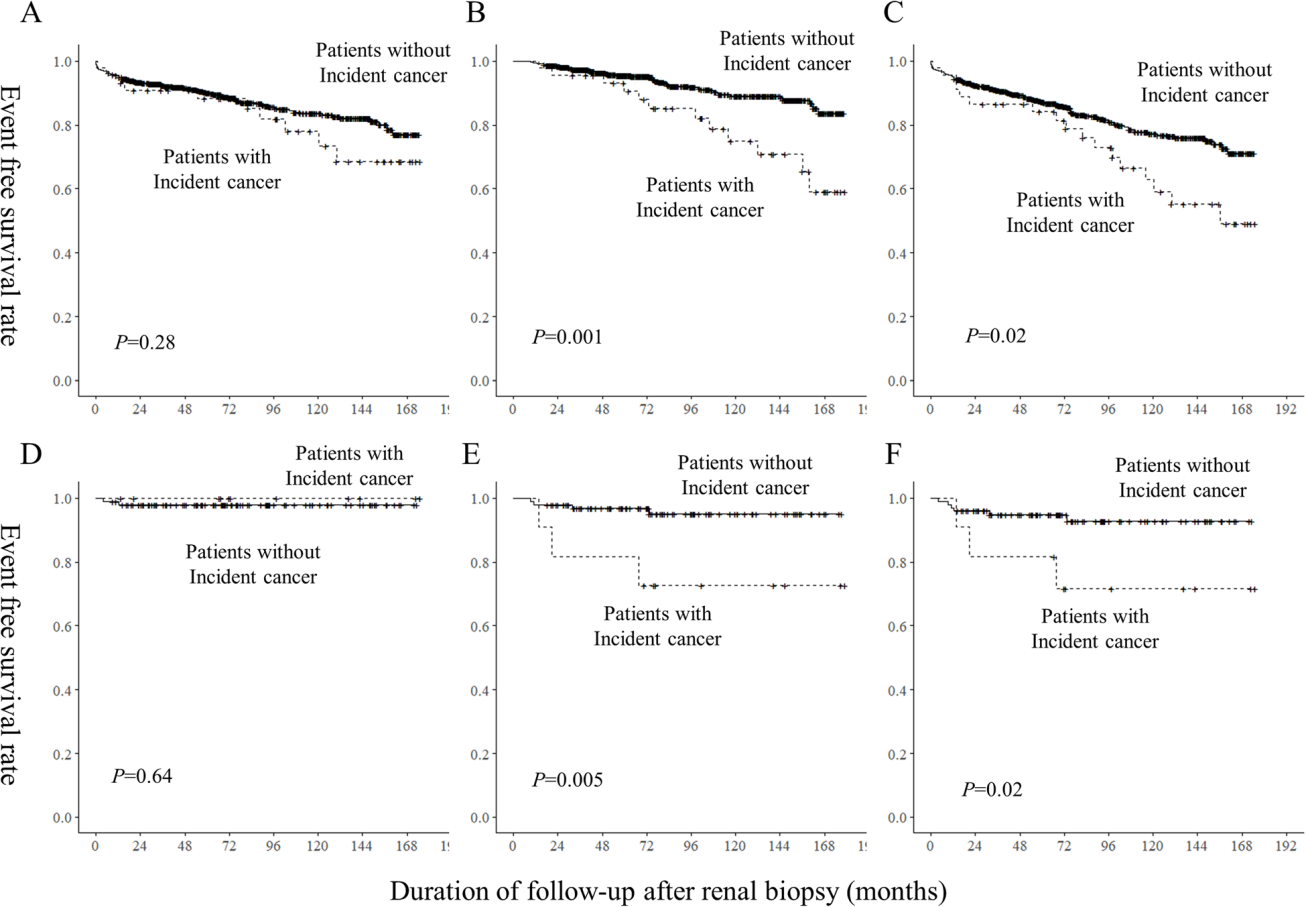
Outcomes of patients according to the development of cancer after renal biopsy. **a**) and **d**) ESRD free-survival rate, **b**) and **e**) Death-free survival rate, and **c** and **f**) Composite event of death- or ESRD-free survival rate. **a**), **b**), and **c**) in all patients and **d**), **e**), and **f**) in patients with membranous nephropathy. *P*-values were calculated by the log-rank test

Sixty-seven subjects died during follow-up, and 106 progressed to ESRD and required renal replacement therapy. A Kaplan–Meier survival curve of ESRD, death, and their composite event were compared according to the presence of de novo cancer development by the log-rank test. Cancer development did not have a significant effect on the ESRD-free survival in the overall cohort and the MN subgroup (*P* = 0.28 and *P* = 0.64, respectively; Fig. 4a and d). However, subjects with cancer had lower death-free and composite event-free survival rates compared to those without cancer in the overall cohort (*P* = 0.001 and *P* = 0.02; Fig. 4b and c) and the MN subgroup (*P* = 0.005 and *P* = 0.02; Fig. 4e and f). In the multivariable Cox proportional hazard model adjusted for possible confounding factors, cancer incidence was not associated with mortality, ESRD, and the composite outcome in the overall cohort (Table S5). Among subjects with MN, however, cancer incidence was associated with a significantly increased risk of death (HR: 6.59, 95% CI: 1.22–35.56, *P* = 0.03), but not with the risk of ESRD or the composite outcome.

## Discussion

Previously, a few studies have reported an increase in the long-term risk of cancer development among patients with GN. A study based on Danish registry data showed an increased risk of cancer development up to 5 years after a GN diagnosis, while another study based on Norwegian registry data observed an increased risk even 5–15 years after an MN diagnosis [10, 17]. Christiansen et al. reported a 73% increase in the 5-year cancer incidence among patients with nephrotic syndrome, compared to the general population [18]. However, these studies only included data from Western populations, focused especially on MN or nephrotic syndrome, and used registry data that lacked specific clinical information (e.g., immunosuppressant usage or clinical outcomes after cancer development). Therefore, we conducted this study to determine the incidence of de novo cancer in a Korean population of patients with GN, including all major types of GN, through a thorough review of clinical data. Moreover, we assessed differences in the outcomes of mortality and ESRD development according to de novo cancer development in our patient cohort. To the best of our knowledge, this is the first report on the incidence and types of de novo cancers in an Asian population with GN. As the types of GN and the types and incidence of cancer differ by race and ethnicity, this study provides valuable insights into the incidence and outcomes of cancer in Asian patients with GN.

In this study, we identified a de novo cancer incidence of 5.5% in a cohort of Korean patients with renal biopsy-confirmed GN during a mean follow-up duration of 4.9 years. Overall, patients with GN had a higher risk of cancer development, with higher SIR, relative to the general population, particularly in GNs such as MN, IgAN, lupus nephritis, and FSGS. These findings definitely suggest that de novo cancer risk in GN patients was substantially higher risk during follow-up after GN diagnosis.

From our results, the most common cancer types were colon cancer, hepatocellular carcinoma, and papillary thyroid carcinoma, all of which are among the six major cancers affecting the general Korean population [21]. These common cancer types differ from those reported in previous reports from Western countries, wherein the greatest increases were observed for non-Hodgkin’s lymphoma and skin cancer in the GN population relative to the general population [10, 17, 22]. For patients with MN, the most common cancer types were reported as lung cancer, prostate cancer, and hematologic malignancy [23], but most of these were diagnosed at the time of or within a short period after MN diagnosis. Bjørneklett et al. showed a significantly increased SIR only for prostate cancer in MN patients during a long-term follow-up period (mean 6.2 years) [24]. We found that the cancer types with increased SIR in patients with MN were hematologic malignancies and non-melanoma skin cancer. These differential results may be attributable to the small number of observed cancer cases and race differences. The findings of the present and previous studies emphasize that screening for de novo cancer during the follow-up period after GN diagnosis should include cancers that occur most often in the corresponding general population. Further studies including larger numbers of cases are needed to confirm whether patients with GN are at an increased risk of specific types of de novo cancer relative to the general population, particularly in Asian countries.

We identified a pathologic diagnosis of MN, old age, and low hemoglobin level as independent risk factors for cancer development in GN patients during the follow-up period. Moreover, in subjects with MN, old age (≥65 years) was identified as an independent risk factor for cancer development, consistent with the findings of a previous study [17]. Additionally, when we analyzed the outcomes of GN patients according to the cancer development status, those with cancer had an increased risk of mortality but not of ESRD development, especially in MN. As previously noted, our data support the emphasis of cancer surveillance for these high-risk subgroups of GN patients. The early detection of de novo cancers and timely intervention would likely improve the survival outcomes of patients with GN.

The pathophysiologic mechanisms underlying the association between GN and long-term cancer development are unclear. Possibly, GN promotes cancer development through the immunosuppressive effects of nephrosis or uremia [22]. Moreover, the carcinogenic effects of GN treatment might induce de novo cancer development. Cyclophosphamide use was associated with a lower cancer-free survival in the Kaplan-Meier analysis, but after adjusting for confounding factors, we did not find a significant effect of the use or dosage of immunosuppressant agents on cancer development. We further note that an underlying undiagnosed tumor may induce GN through mechanisms such as antigen production and might be detected as de novo cancer during follow-up [10]. Moreover, infection with a viral pathogen, such as hepatitis B virus, may be an etiological factor in both GN and cancer [25]. We note that GN patients are monitored and closely followed by nephrologists, which may lead to a higher cancer detection rate than that in the general population. Additional studies are required to elucidate the physiologic mechanism underlying the association between GN and de novo cancer.

This study has several limitations due to the nature of a retrospective cohort study. First, although we excluded patients who developed cancer within 6 months after diagnosis, patients with hidden underlying malignancies might still have been included. Second, we were unable to determine the specific mechanism underlying the association between GN and de novo cancer from these data. Third, the relatively small number of patients, particularly those with cancer, might reduce the statistical power of our findings. Fourth, there is a lack of information on the results of serologic test for the major target antigens in idiopathic MN such as phospholipase A2 receptor and thrombospondin type-1 domain-containing 7A (THSD7A) [26, 27]. Particularly, THSD7A was found to be closely associated with the occurrence of malignancy [28]. Data on THSD7A antibodies might be helpful to evaluate cancer development in MN patients in future studies.

## Conclusions

This study presents the first analysis of the de novo cancer incidence in an Asian population of patients with GN and demonstrated an increased risk of cancer development in Korean GN patients as reported in a Western population. We identified old age and pathologic diagnosis of MN as independent risk factors for de novo cancer development after adjusting for confounding factors. Furthermore, we showed an increase in all-cause mortality among patients with GN patients who developed cancer, particularly in the subgroup of patients with MN. Our results suggest that GN patients without concurrent cancer should be made aware of the risk of subsequent de novo cancer development. In particular, patients with MN should be screened carefully, as they face significantly higher risks of cancer development and associated mortality. More studies are needed to determine the specific cancer types associated with GN and the underlying pathophysiological mechanisms. Our findings provide the basis for changes in cancer surveillance guidelines in the GN population and result in improved outcomes.

## Supplementary information

**Additional file 1 Figure S1.** Cancer incidence after renal biopsy during the follow-up period; **Figure S2.** Kaplan-Meier survival curves for cancer-free survival according to the usage of cyclosporine, tacrolimus, mycophenolate, and azathioprine. **Table S1.** Standardized incidence ratio of de novo cancers in the cohort from 2003 to 2017; **Table S2.** Associations of pathologic diagnosis with cancer development; **Table S3**. Standardized incidence ratios of different cancer types in patients with membranous nephropathy compared with the age- and sex-matched general population; **Table S4.** Risk factor to develop cancer in membranous nephropathy patients; **Table S5.** Effects of cancer incidence on outcomes in all patients and in patients with membranous nephropathy.

## Data Availability

The datasets used and/or analyzed during the current study are available from the corresponding author on reasonable request.

## Abbreviations

GN: Glomerulonephritis
ESRD: End-stage renal disease
MN: Membranous nephropathy
CI: Confidence interval
MCD: Minimal change diseases
MPGN: Membranoproliferative glomerulonephropathy
FSGS: Focal segmental glomerulosclerosis
IgAN: Immunoglobulin A nephropathy
TIN: Tubulointerstital injury
TMA: Thrombogenic microangiopathy
eGFR: Estimated glomerular filtration rate
SIR: Standardized incidence ratio
