# Supplementary material for: Cancer development and mortality differences in patients with glomerulonephritis after renal biopsy: a single center retrospective cohort study
Source: BMC Nephrol. 2020 Jun 10;21:221. doi: 10.1186/s12882-020-01882-x (PMC7288504; doi:10.1186/s12882-020-01882-x)
Supplement: Supplementary file 1 — Additional file 1 Figure S1. Cancer incidence after renal biopsy during the follow-up period; Figure S2. Kaplan-Meier survival curves for cancer-free survival according to the usage of cyclosporine, tacrolimus, mycophenolate, and azathioprine. Table S1. Standardized incidence ratio of de novo cancers in the cohort from 2003 to 2017; Table S2. Associations of pathologic diagnosis with cancer development; Table S3. Standardized incidence ratios of different cancer types in patients with membranous nephropathy compared with the age- and sex-matched general population; Table S4. Risk factor to develop cancer in membranous nephropathy patients; Table S5. Effects of cancer incidence on outcomes in all patients and in patients with membranous nephropathy. [file 12882_2020_1882_MOESM1_ESM.docx]

**Figure S1.** Cancer incidence after renal biopsy during the follow-up period.


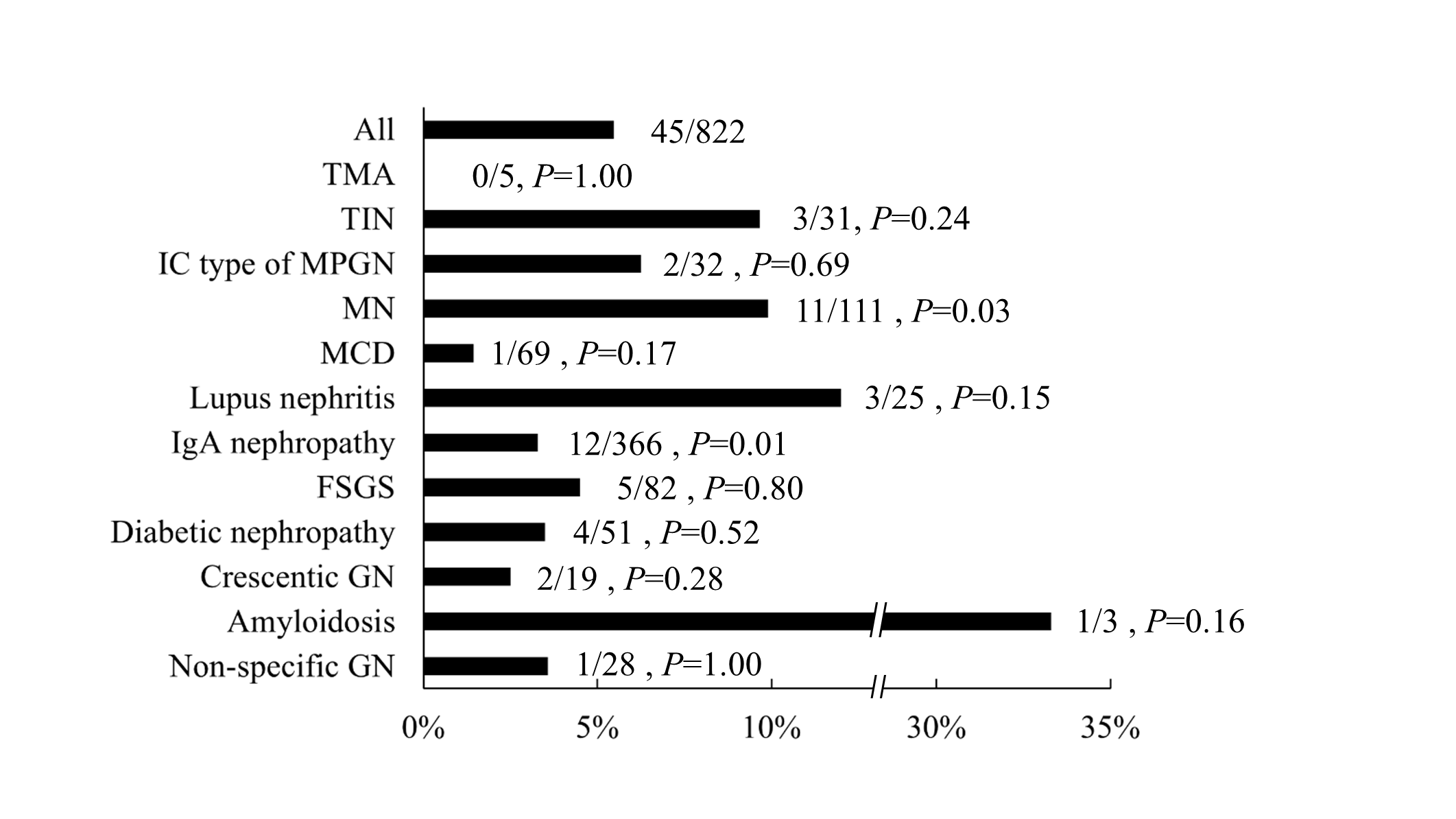


Mean follow-up months until cancer development from renal biopsy was 58.9 ± 44.5 months. *P*-value was compared the incidence of cancer between a specific diagnosis and the others by Pearson’s Chi-square test or Fisher’s exact test according to the number of subgroups. FSGS, focal segmental glomerulosclerosis; GN, Glomerulonephritis; IC, immunocomplex; MCD, minimal change disease; MN, membranous nephropathy; MPGN, membranoproliferative glomerulonephritis; TIN, tubulointerstitial injury; TMA, thrombogenic microangiopathy.

**Figure S2.** Kaplan-Meier survival curves for cancer-free survival according to the usage of cyclosporine (A), tacrolimus (B), mycophenolate (C), and azathioprine (D).


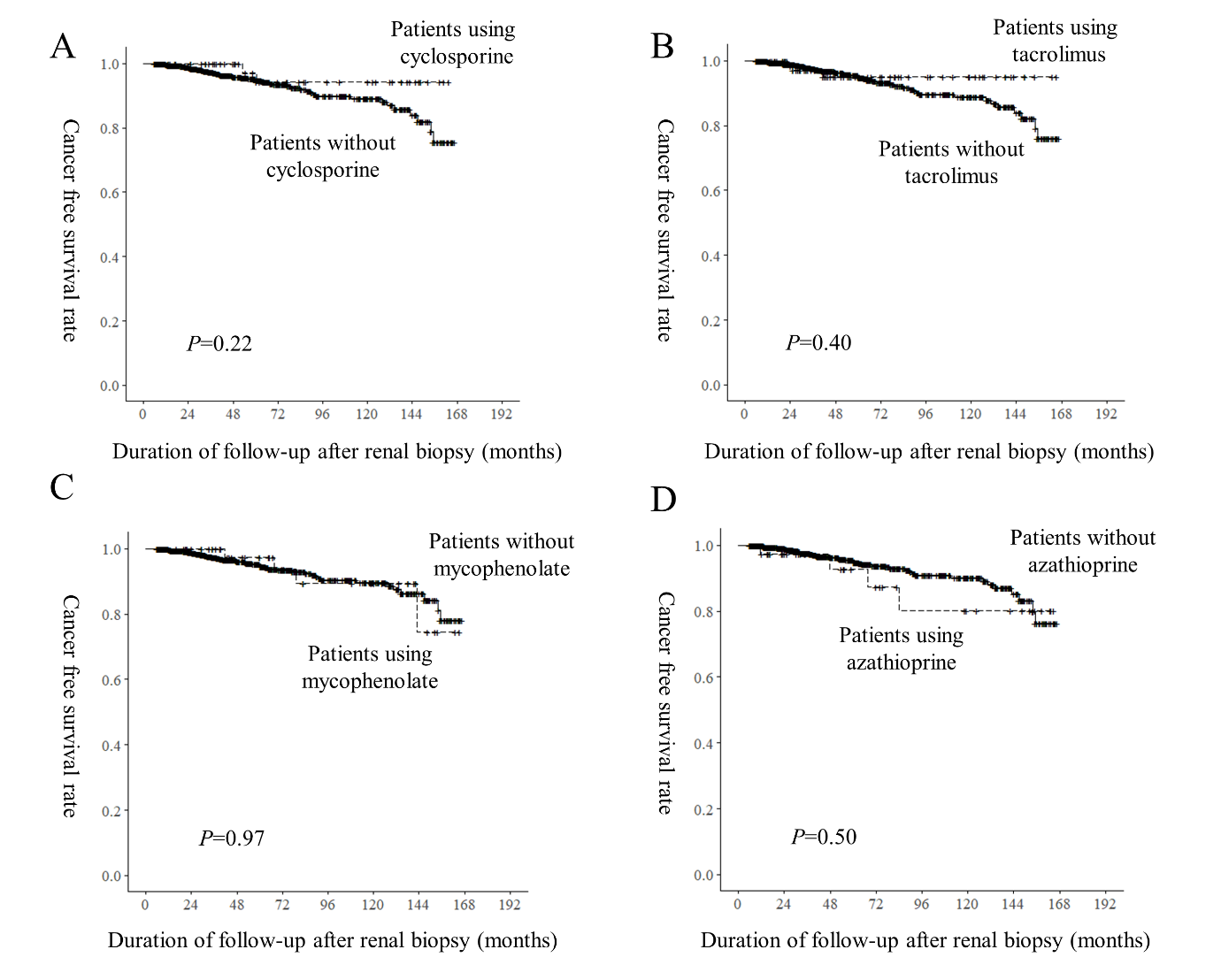


**Table S1.** Standardized incidence ratio of de novo cancers in the cohort compared with the age- and sex-matched Korean general population from 2003 to 2017

|  |  | Number  of study patients | Observed  Cancer incidence | Expected  Cancer incidence | Age- and sex-standardized incidence ratio (95% CI) |
| --- | --- | --- | --- | --- | --- |
| All patients* | | 822 | 45 | 6.29 | 7.16 (5.22-9.61) |
| Pathologic diagnosis | Non-specific GN | 28 | 1 | 0.20 | 5.06 (0.07-41.25) |
|  | Amyloidosis | 3 | 1 | 0.05 | 20.06 (0.26-163.63) |
|  | Crescentic GN* | 19 | 2 | 0.20 | 10.06 (1.13-43.68) |
|  | Diabetic nephropathy* | 51 | 4 | 0.48 | 8.31 (2.24-23.07) |
|  | FSGS* | 82 | 5 | 0.65 | 7.73 (2.49-19.17) |
|  | IgA nephropathy* | 366 | 12 | 1.98 | 6.05 (3.12-10.77) |
|  | Lupus nephritis* | 25 | 3 | 0.08 | 39.46 (7.93-129.29) |
|  | MCD | 69 | 1 | 0.65 | 1.54 (0.02-12.56) |
|  | MN* | 111 | 11 | 1.23 | 8.92 (4.44-16.30) |
|  | MPGN | 32 | 2 | 0.40 | 5.06 (0.57-21.97) |
|  | TIN* | 31 | 3 | 0.34 | 8.82 (1.77-28.89) |
|  | TMA | 5 | 0 | 0.04 | 0 (uc) |

^§^Age at cancer diagnosis. *Statistically significant. uc: unable to calculate. GN, Glomerulonephritis; FSGS, focal segmental glomerulonephritis; IgAN, IgA nephropathy; MCD, minimal change disease; MN, membranous nephropathy; MPGN, membranoproliferative glomerulonephritis; TIN, tubulointerstitial nephropathy; TMA, thrombotic microangiopathy.

**Table S2.** Associations of pathologic diagnosis with cancer development.

| Pathologic findings | Univariable analysis | |  | Multivariable anlaysis* | |
| --- | --- | --- | --- | --- | --- |
|  | HR (95% CI) | *P*-value |  | HR (95% CI) | *P*-value |
| Amyloidosis (presence) | 7.76 (1.06-57.05) | 0.04 |  | 5.41 (0.69-42.61) | 0.11 |
| Crescentic GN (presence) | 1.59 (0.38-6.57) | 0.53 |  | 0.47 (0.10-2.21) | 0.34 |
| DM nephropathy (presence) | 2.50 (0.89-7.05) | 0.08 |  | 1.24 (0.33-4.68) | 0.75 |
| FSGS (presence) | 0.90 (0.36-2.29) | 0.83 |  | 1.41 (0.21-2.39) | 0.58 |
| IgAN (presence) | 0.43 (0.22-0.82) | 0.01 |  | 0.79 (0.36-1.75) | 0.56 |
| Lupus nephritis (presence) | 1.43 (0.44-4.68) | 0.55 |  | 2.86 (0.68-11.98) | 0.15 |
| MCD (presence) | 0.33 (0.04-2.37) | 0.27 |  | 0.37 (0.05-2.84) | 0.34 |
| MN (presence) | 2.27 (1.15-4.49) | 0.02 |  | 2.30 (1.06-4.98) | 0.03 |
| MPGN (presence) | 1.08 (0.26-4.48) | 0.91 |  | 0.46 (0.10-2.09) | 0.31 |
| TIN (presence) | 2.89 (0.89-9.40) | 0.08 |  | 1.38 (0.38-4.99) | 0.62 |

*Adjusted with age, gender, and clinical parameters related to incident cancer, such as diabetes mellitus, coronary heart disease, smoking status, chronic hepatitis B and C, liver cirrhosis, levels of hemoglobin and serum creatinine at renal biopsy, usage of each immunosuppressive agent such as azathioprine, cyclophosphamide, cyclosporin, mycophenolate, steroid, rituximab, and tacrolimus after renal biopsy but before the development of cancer.

**Table S3.** Standardized incidence ratios of different cancer types in patients with membranous nephropathy compared with the age- and sex-matched general population

| Site of cancers | Expected  Cancer incidence | Sex and age-standardized incidence ratio | 95% CI | |
| --- | --- | --- | --- | --- |
| All cancers* | 1.23 | 8.92 | 4.44 | 16.30 |
| Squamous cell carcinoma of skin* | 0.03 | 79.02 | 8.87 | 342.93 |
| Acute myelocytic leukemia* | 0.01 | 117.69 | 1.54 | 960.09 |
| Hepatocellular carcinoma | 0.12 | 8.43 | 0.11 | 68.74 |
| Stomach cancer | 0.22 | 4.55 | 0.06 | 37.11 |
| Bladder cancer | 0.03 | 29.30 | 0.38 | 239.02 |
| Prostate cancer | 0.08 | 13.04 | 0.17 | 106.41 |
| Multiple myeloma* | 0.01 | 131.15 | 1.71 | 1069.95 |
| Lymphoma | 0.02 | 50.51 | 0.66 | 412.06 |
| Colon cancer | 0.08 | 12.85 | 0.17 | 104.84 |
| Lung cancer | 0.19 | 5.16 | 0.07 | 42.13 |

**Table S4.** Risk factor to develop cancer in membranous nephropathy patients

| Variables | B | Wald | Hazard ratio | 95% Confidence interval | | *P*-value |
| --- | --- | --- | --- | --- | --- | --- |
| Age (per 10 years) | 0.74 | 6.69 | 2.10 | 1.20 | 3.69 | 0.01 |
| Age group* |  |  |  |  |  |  |
| <65 year |  |  | Reference | | | |
| ≥65 year | 2.03 | 6.29 | 7.61 | 1.56 | 37.16 | 0.01 |

*Age group: compared to patients aged <65 years. Model: Cox's hazard proportional model adjusted with clinical findings at renal biopsy, such as age, gender, level of serum sodium and hemoglobin, and pathologic findings of global sclerosis and presence of electron dense deposit on mesangium.

**Table S5.** Effects of cancer incidence on outcomes in all patients and in patients with membranous nephropathy.

| Patients' group | Outcomes | Hazard ratio | 95% Confidence interval | | *P*-value |
| --- | --- | --- | --- | --- | --- |
| All patients | For end-stage renal disease^1^ | uc | uc | uc | 0.62 |
|  | For death ^2^ | uc | uc | uc | 0.70 |
|  | For composite outcome^3^ | uc | uc | uc | 0.72 |
| Patients with MN | For end-stage renal disease^1^ | uc | uc | uc | 0.77 |
|  | For death^2^ | 6.59 | 1.22 | 35.56 | 0.03 |
|  | For composite outcome^3^ | uc | uc | uc | 0.19 |

uc: unable to calculate.

^1^ Cox's hazard proportional model adjusted with clinical findings at renal biopsy, such as age, gender, history of coronary artery disease, cerebrovascular disease, diabetes mellitus, and hypertension, systolic blood pressure, diastolic blood pressure, pathologic diagnosis of renal disease, and laboratory findings of albumin, cholesterol, hemoglobin, and eGFR, and development of cancer during the follow-up period.

^2^ Cox's hazard proportional model adjusted with clinical findings at renal biopsy, such as age, gender, history of coronary artery disease, cerebrovascular disease, diabetes mellitus, and hypertension, systolic blood pressure, pathologic diagnosis of renal disease, and laboratory findings of hemoglobin and eGFR, and development of cancer during the follow-up period.

^3^ Cox's hazard proportional model adjusted with clinical findings at renal biopsy, such as age, gender, history of coronary artery disease, cerebrovascular disease, diabetes mellitus, and hypertension, systolic blood pressure, pathologic diagnosis of renal disease, and laboratory findings of hemoglobin and eGFR, and development of cancer during the follow-up period.
